# Neurons in the inferior colliculus use multiplexing to encode features of frequency-modulated sweeps

**DOI:** 10.1101/2025.02.10.637492

**Published:** 2025-02-10

**Authors:** Audrey C. Drotos, Sarah Z. Wajdi, Michael Malina, Marina A. Silveira, Ross S. Williamson, Michael T. Roberts

**Affiliations:** 1. Kresge Hearing Research Institute, Department of Otolaryngology – Head and Neck Surgery, University of Michigan, Ann Arbor, Michigan 48109; 2. Department of Molecular and Integrative Physiology, University of Michigan, Ann Arbor, Michigan 48109; 3. Departments of Otolaryngology-Head & Neck Surgery and Neurobiology, University of Pittsburgh, PA, 16260; 4. Neuroscience Institute, Carnegie Mellon University, Pittsburgh, PA 15213; 5. Department of Neuroscience, Development and Regenerative Biology, University of Texas at San Antonio, San Antonio, Texas, 78249

## Abstract

Within the central auditory pathway, the inferior colliculus (IC) is a critical integration center for ascending sound information. Previous studies have shown that many IC neurons exhibit receptive fields for individual features of auditory stimuli, such as sound frequency, intensity, and location, but growing evidence suggests that some IC neurons may multiplex features of sound. Here, we used in vivo juxtacellular recordings in awake, head-fixed mice to examine how IC neurons responded to frequency-modulated sweeps that varied in speed, direction, intensity, and frequency range. We then applied machine learning methods to determine how individual IC neurons encode features of FM sweeps. We found that individual IC neurons multiplex FM sweep features using various strategies including spike timing, distribution of inter-spike intervals, and first spike latency. In addition, we found that decoding accuracy for sweep direction can vary with sweep speed and frequency range, suggesting the presence of mixed selectivity in single neurons. Accordingly, using static receptive fields for direction alone yielded poor predictions of neuron responses to vocalizations that contain simple frequency changes. Lastly, we showed that encoding strategies varied across individual neurons, resulting in a highly informative population response for FM sweep features. Together, our results suggest that multiplexing sound features is a common mechanism used by IC neurons to represent complex sounds.

## Introduction

One of the primary functions of the brain is to build representations of complex sensory objects based on the relatively limited set of features encoded by the sensory periphery. Many previous studies have shown that neurons in the inferior colliculus (IC), the midbrain hub of the central auditory pathway, can exhibit receptive fields for specific sound features. For example, IC neuron firing rate can be modulated by the frequency and intensity of pure tone stimuli (Suga, 1964; Ramachandran et al., 1999; Palmer et al., 2013), and IC neurons can exhibit firing-rate based tuning for sound duration (Casseday et al., 1994; Ehrlich et al., 1997; Fuzessery and Hall, 1999; Pérez-González et al., 2006) and sound location (Aitkin et al., 1984; Kuwada et al., 1987; Davis et al., 2003; Chase, 2005; Slee and Young, 2011). Prior studies also suggest that some IC neurons use changes in firing rate to encode the direction of rapid movements across sound frequency known as frequency-modulated (FM) sweeps (Suga, 1965; Poon et al., 1991; Fuzessery, 1994; Fuzessery et al., 2006; Andoni et al., 2007; Xie et al., 2007; Gittelman et al., 2009; Kuo and Wu, 2012; Geis and Borst, 2013).

However, individual neurons have many available strategies by which they can encode sound features. In addition to firing rate, neurons can use the temporal patterning of spikes, including inter-spike interval distributions and/or first spike latency, to transmit information about incoming stimuli to postsynaptic partners. In addition, auditory neurons can use these varying strategies to encode multiple sound features at once in a process called multiplexing, where one neuron uses two or more coding strategies to simultaneously convey information about different aspects of a stimulus. Evidence for multiplexing in auditory neurons has been found in auditory cortex (Furukawa and Middlebrooks, 2002; Lakatos et al., 2007; Walker et al., 2011; Insanally et al., 2019), and growing evidence suggests it also exists in IC (Zheng and Escabí, 2008; Day and Delgutte, 2016; Caruso et al., 2018). For example, the spiking patterns of single IC neurons can carry information about three sound localization cues (Chase and Young, 2008), and IC neurons can also co-encode information about sound and task-relevant variables in a behavioral task (Quass et al., 2024). In addition, IC neurons can simultaneously encode information across sensory modalities, including visual and auditory stimuli (Schmehl et al., 2025). Multiplexing thus provides a way for individual IC neurons to encode multiple features of a complex sound stimulus.

FM sweeps are are common components of natural sounds, including mouse vocalizations (Doupe and Kuhl, 1999; Luo et al., 2007; Portfors et al., 2009). In addition, some IC neurons exhibit mixed selectivity for features of FM sweeps where the neuron response depends on the co-occurrence of multiple sound features, such as sweep rate and direction (Andoni et al., 2007; Andoni and Pollak, 2011). However, whether and how mouse IC neurons simultaneously encode multiple features of FM sweeps has not been well studied.

Here, we performed in vivo, juxtacellular recordings from IC neurons in awake mice and used machine learning to examine whether and how single cells and populations of IC neurons encode features of FM sweeps. We first found that sweep direction is encoded not only by the neuron firing rate but also by other temporal dimensions of the neuron response. In addition, we found that IC neurons exhibit mixed selectivity in response to FM sweep features, such that receptive fields for FM sweep direction are dependent on sweep speed and frequency range. Sweep features could also be decoded from distinct parameterizations of spiking in individual neurons, revealing that cells use multiple strategies to encode stimulus-specific information. In addition, we found that multiple features could be decoded from individual IC neurons, indicating that cells multiplex sound features. While IC neurons exhibited heterogeneous responses at the single-cell level, we found that decoding from populations of neurons increased the accuracy of the model, highlighting a role for population-level codes in the IC. Overall, we found that individual IC neurons can multiplex FM sweep features, highlighting a method that IC neurons use to build representations of complex sensory objects.

## Materials and Methods

### Animals

All experiments were approved by the University of Michigan Institutional Animal Care and Use Committee and were in accordance with NIH guidelines for the care and use of laboratory animals. Animals were given continuous access to food and water and stayed on a 12-hour day/night cycle throughout their care and use. Mice on a CBA/CaJ or CBA/CaJ × C57BL/6J background of both sexes were used for this study. Mice on a CBA/CaJ background were used because mice on the C57BL/6J background have a single-nucleotide polymorphism in the *Cdh23* gene which impairs inner ear hair cell function and causes age-related hearing loss (Noben-Trauth et al., 2003). In addition to their background, mice used in the study were age P61-P86, and animals this age range do not exhibit age-related hearing loss on auditory brainstem response tests (Kane et al., 2012). Additionally, neural thresholds for 200 ms pure tones between 4 and 64 kHz were evaluated and all neurons with V-shaped receptive fields had thresholds at 10 dB, the lowest sound level tested.

### Headbar implantation

Mice were anesthetized using 1–3% isoflurane (Piramal Critical Care, # NDC 66794-017-25), and body temperature was maintained using a homeothermic heating pad placed under the mouse. To reduce post-operative pain, mice were given subcutaneous injections of carprofen (5 mg/kg, CarproJect, Henry Schein Animal Health) and buprenorphine (0.1 mg/kg). Hair on the scalp was removed using scissors, and a circular portion of scalp approximately 11–12 mm in diameter was removed to expose the skull. The inferior colliculus was identified using stereotaxic coordinates (relative to lambda and in μm: 900 caudal, 1125 lateral) and marked using a surgical marker. Bupivacaine (0.2 mL) was then applied to numb the skull, and the periosteum was removed and the skull was thoroughly scored using a #11 scalpel. To begin adhesive preparation for the headbar, a thin layer of dental acrylic (C&B Metabond from Parkell, cat #s S398, S399, S371) was applied using a fine paintbrush, which was then left to cure for ten minutes. On top, a thin layer of dental acrylic resin (Henry Schein, Inc., # 1259208 and # 1250143) was applied. Dental acrylic resin was also applied to a custom-designed headbar that was then placed onto the skull. To secure the headbar to the skull, dental acrylic resin was added in layers to cover the top of the headbar, and then silicone elastomer (Smooth-On Inc. Body Double FAST) was placed in the opening over the skull and secured with a thin strip of dental acrylic resin. Mice were monitored for signs of pain or distress for one hour post-procedure and were ambulatory before being returned to the vivarium. An additional injection of carprofen was administered the day following surgery for postoperative analgesia, and animals continued to be monitored for signs of pain or distress for 7 days.

### Craniotomy surgery

Prior to the first day of recording, mice were anesthetized using 1–3% isoflurane. Body temperature was maintained using a homeothermic heating pad placed under the mouse. To reduce post-operative pain, mice were given a subcutaneous injection of carprofen (5 mg/kg, CarproJect). Using the IC mark that was made during the headbar implantation procedure, a craniotomy was made using intervals of drilling with a micromotor drill (K.1050, Foredom Electric Co.) with a 0.5 mm burr (Fine Science Tools). Drilling began with shallow and small circles and was interrupted every ~20 seconds with application of cold PBS to ensure brain temperature was unaffected. When the skull above the IC was thin enough, a #11 scalpel and/or 27-gauge needle was used to remove a small section of skull above the IC. For one hour post-procedure, mice were monitored for signals of pain or distress.

### In vivo electrophysiology recordings

29 mice (14 female, 15 male) aged P61-P86 at the time of first recording were used for the study. Mice began habituation to the head fixation setup 3 or more days following headbar implantation to allow for recovery. Habituation consisted of one day of handling within the mouse’s cage and 3–5 days of head fixation inside the sound isolation booth, with the duration of head fixation increasing every day until the mouse tolerated 80 minutes of head fixation (10 minutes, 20 minutes, 40 minutes, 60 minutes, 80 minutes). A craniotomy was then surgically drilled above the left IC using the procedures outlined here under “Craniotomy”. After one hour of recovery, mice were placed in the head fixation setup. The recording pipette was backfilled with an in vivo internal solution (in mM, 135 NaCl, 5.4 KCl, 1.5 CaCl_2_, 1 MgCl_2_, 5 HEPES, pH set to 7.3 with NaOH). An MP-285 micromanipulator (Sutter Instruments) was used to slowly advance the pipette into the IC, and neurons were identified for juxtacellular patch-clamp recordings by an increase in electrode resistance and the presence of action potentials. Once a loose seal was made with a cell (6–47 MΩ), a regularly calibrated, free-field electrostatic speaker (Tucker-Davis Technologies, ES1) was used to present sound stimuli to the mice. The speaker was positioned contralateral to the IC recording site, at ~10 cm in front of the right ear, at a 45° angle. Sound stimuli consisted of 100 ms white noise bursts (4–64 kHz, 70 dB SPL), various stimuli used to test IC responses to frequencies under different conditions (outlined in Table 1), and various mouse vocalizations presented forwards and backwards. Data were acquired at 50 kHz using a Dagan amplifier, an NI PCIe-6343 data acquisition board, and the program WaveSurfer (Janelia) via MATLAB. Individual mice were recorded from once daily for fewer than 10 sessions total. Recording locations were verified post-hoc by dissection to visualize the electrode penetration tract and/or labeling with the fluorescent marker DiI (ThermoFisher Scientific, #V22885).

### Analysis of electrophysiological recordings

Electrophysiology data were acquired and high-pass filtered at 300 Hz using MATLAB (Mathworks, 2022a-2024a). Data were re-associated with stimuli information and metadata post-collection using a custom MATLAB script, and spikes were detected using a threshold crossing. Spike detection was validated by performing k-means clustering on the amplitude and full-width at half-maximum amplitude of each spike; recordings that exhibited multiple clusters using this analysis were excluded from the dataset. Frequency response area plots were made using the ‘imagesc’ function in MATLAB.

### Machine learning models

Analyses were performed in Python 3.11.9. After data pre-processing (outlined above), electrophysiology data were saved into a structure and imported to Python. Support vector machine (SVM) models were implemented in Python using the ‘SVC’ function with a linear kernel from the ‘sklearn’ package. The C parameter was set to 1 for all models. The model features were the binned spike times from each trial, and the number of bins was determined by examining model performance across 1–501 bins (steps of 2) for decoding direction of the FM sweep. The resulting bin sizes are shown in Table 2. To maintain consistency across each model, we used the bin size that resulted in the highest accuracy (2.3 ms) as model inputs for each stimulus. The resulting number of bins for each stimulus is shown in Table 2. The total time binned for each stimulus corresponded to the longest duration FM sweep for that stimulus set plus 20% of the stimulus time to account for time delay for sound to reach the IC and any offset spiking that might contribute to coding.

After binning, models were trained on 80% of the data and tested on 20% of the data using 5-fold cross validation. In cases where direct comparisons were made between the sound feature decoding accuracy scores in the same neuron (for different sound features), the data were downsampled to the sound feature with the lowest number of observations and the average k-fold cross validated accuracy from each of 100 resamples was averaged and used. For example, for the 1 octave FM sweep stimulus, we played 100 sweeps/direction and 20 sweeps/speed at each frequency range, resulting in a total of 800 trials (400 per direction, 80 per speed, 100 per frequency range). Therefore, when we performed decoding analyses for frequency range, speed, and direction in the one-octave sweep condition, we used 80 trials/feature to decode each class (direction, speed, frequency range). Plots from SVM analyses were made using the ‘matplotlib’ and ‘seaborn’ packages in Python, and accuracy scores for points on the same axes are always directly comparable (had the same number of trials/class in the model).

To determine if the decoding model performed above chance in individual neurons, accuracy scores for true data were compared to accuracy scores for the model tested and trained on data with shuffled class labels. To obtain the accuracy scores for shuffled data, class labels were shuffled 100 times and the SVM was fit to the shuffled data each time. The mean and standard deviation from the distribution of shuffled accuracies was calculated for each cell. The z score for the actual model accuracy was calculated with reference to the shuffled distribution and the accuracy of the model was considered above chance if this value exceeded 1.96, (the 95% confidence interval of a standard distribution with μ = 1, σ = 1).

### Comparison of decoding from spike parameterizations

To compare decoding accuracy for FM sweep features across varying spike parametrizations, we calculated the distribution of the inter-spike intervals and first spike latency for each trial. To calculate the inter-spike interval, we calculated the distance between each of the spikes and binned this distribution using 100 bins, meaning the model inputs were 100 features. To calculate the first spike latency, we assumed a 5 ms delay to the IC (Felix et al., 2019) and used the first spike that occurred 5 ms after the stimulus began.

### Pseudo-population decoding

To examine how groups of IC neurons encode complex sound features, we evaluated decoding accuracy in pseudo-populations of IC neurons consisting of 2–20 cells. First, we examined coding in an individual neuron, as described above. Next, we randomly sampled 10 neurons from our population and paired each of these neurons individually with the first neuron. To evaluate decoding for neuron pairs, we horizontally concatenated the binned spike counts (rows) for each neuron pair for trials with matched stimuli and then evaluated the performance of the model on the concatenated data. To find the overall accuracy for the individual cell plus a paired cell, we averaged the model accuracy for each pairing. We repeated this process with every cell in our dataset, pairing it with 10 randomly selected neurons and evaluating average model accuracy across the 10 pairs to get an overall paired accuracy for the population.

We next continued this process by increasing the number of paired cells for each individual neuron the dataset. For each individual cell, there were 10 randomly selected groups of neurons that were paired (e.g., 10 groups of 2, 3, 4, 5… etc.) and decoding was performed on the concatenated binned spike times from these groups. For each cell, the average accuracy from each set of pairings was calculated, and then the average accuracy across the dataset was plotted.

### Statistical tests

All statistical tests were performed using Python 3.11.9. To evaluate whether there was significant decoding accuracy in individual neurons, we z-scored accuracies relative to shuffled class accuracy distributions, as described in section above. To evaluate whether there were differences in decoding accuracy between true and shuffled bin conditions, data were evaluated using a paired t-test (‘ttest_rel’ function from ‘scipy.stats’). To examine correlations between decoding for sound features, linear regression was performed using the ‘linregress’ function from ‘scipy.stats’.

To examine direction decoding accuracy across other varying sweep parameters such as sweep intensity and speed, a repeated-measures ANOVA (‘ANOVArm’ from ‘statsmodels.stats.anova’) was performed followed by Tukey’s HSD post-hoc tests (‘pairwise_tukeyhsd’ from ‘statsmodesl.stats.multicomp’) where appropriate. We also used a repeated-measures ANOVA and Tukey’s HSD post-hoc test to examine if increasing the number of cells in each pseudo-population increased the average accuracy. To test for an increase in the number of cells that significantly encoded direction over increasing speed or intensity, we used a Chi-square test for trend (‘chi2_contingency’ from ‘scipy.stats’), which can be used to examine changes in proportions over ordered groups.

All p-values were compared to a Bonferroni-corrected α value for evaluation of significance where appropriate, and in some cases Bonferroni-adjusted p-values are instead displayed and noted as “adj. p-value”.

### Direction selectivity index calculation

The direction selectivity index (DSI) was calculated as follows:

$$\text{DSI}=\frac{{\text{spikes}}_{\text{up}}-{\text{spikes}}_{\text{down}}}{{\text{spikes}}_{\text{up}}{+\text{spikes}}_{\text{down}}}$$

The time window across which spikes were summed for the 4-octave frequency sweeps was 480 ms after stimulus onset (since the longest stimulus was 400 ms, this is stimulus time + 20% of stimulus time to account for any offset spiking that may be directionally selective). This is the same time window over which spike times were binned for the machine learning analysis.

## Results

### Neurons in awake IC use spike timing to encode the direction of four-octave FM sweeps

**Figure 1.**
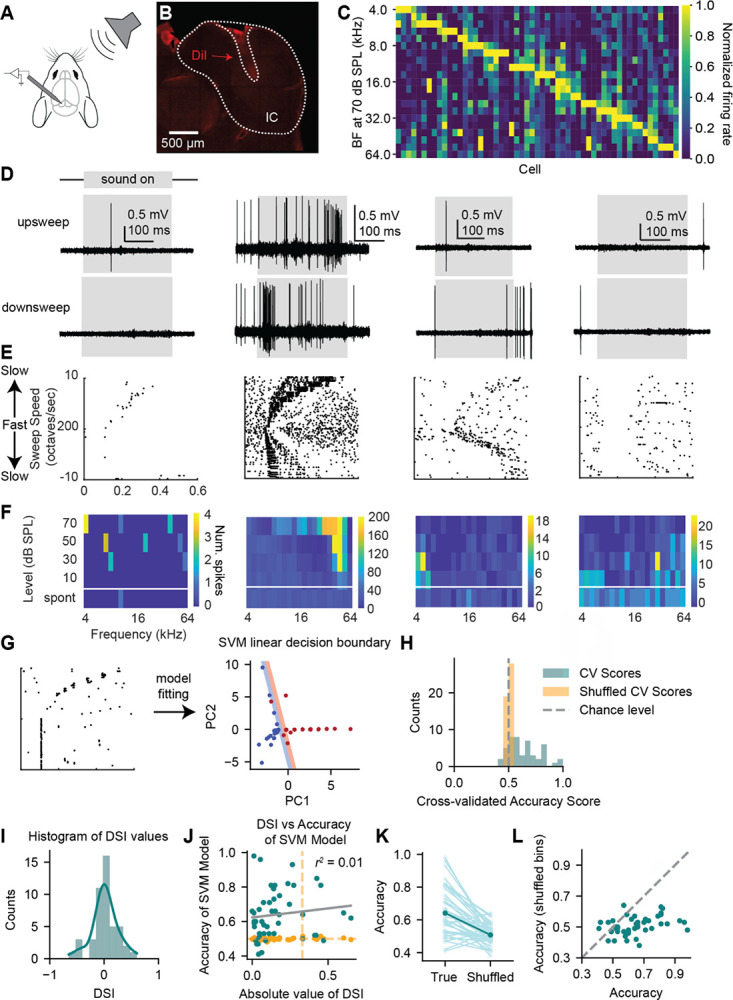
IC neurons use spike timing to encode direction information for FM sweeps ***A***, Experimental design. Awake mice were headfixed in a sound booth with sound presented to the right side of the mouse while juxtacellular recordings were performed in the left IC. ***B***, Example of a DiI labeled recording site in the left IC, outlined in white. ***C***, The tonal receptive field at 70 dB SPL for each neuron included in the study, sorted by best frequency. ***D***, Four example neuron responses to the slowest (10 octaves/s) up (top row) and down (bottom row) frequency-modulated (FM) sweeps. Grey boxes indicate when the sound was presented. ***E***, Raster plots showing responses to four-octave FM sweeps for neurons shown in *D*. The top half of the plot shows responses to upward sweeps, and the bottom half of the plot shows responses to downward sweeps. Sweep speed varies along the y-axis with the fastest sweep speed shown at the middle of the y-axis (200 octaves/s) and sweep speeds becoming progressively slower toward the top and bottom of the y-axis. ***F***, Frequency response areas (FRAs) for neurons shown in *D*. FRAs were constructed using 200 ms tone pips presented in pseudorandom order to cover a 4–64 kHz frequency range at 5 steps/octave intervals and the listed intensities. The bottom row of the heatmap indicates the spontaneous firing rate of the neuron. ***G***, Example of a hyperplane from a model trained to decode direction from neural spike time data. For visualization purposes, data were binned into 16 bins and then PCA-transformed and projected onto the first two principal components. Colored regions on each side of the hyperplane indicate the margins. ***H***, Direction decoding accuracy scores for 4–64 kHz FM sweeps. Blue bars indicate accuracy scores for model trained on true data; orange bars indicate accuracy for model trained on data with shuffled labels. ***I***, Histogram of direction selectivity indices (DSIs) calculated from the same group of cells shown in *H*. The histogram is overlaid with a kernel density estimate that was generated using the ‘kde’ argument for seaborn’s ‘histplot’ function. ***J***, Scatterplot comparing the accuracy of the model for decoding direction and the absolute value of the DSI in individual neurons. Blue dots are true accuracy, orange dots are accuracy of the model with shuffled class labels. Grey line shows the linear regression between true accuracy labels and DSI, indicating no correlation between the two values. Vertical orange line is the DSI cutoff from Kuo and Wu (2012); neurons to the right of this line are considered direction selective according to the DSI metric. Horizontal orange line is model chance. ***K***, Comparison of model accuracy for direction decoding with time bins in correct order vs. shuffled time bins. Class labels are maintained in each case. Individual neurons are connected by lines, and the mean is shown in dark blue. ***L***, Scatterplot showing the accuracy for true bin order vs. shuffled bin order (same as *K*). The dashed grey line indicates the line of identity (x = y).

Many previous studies quantified direction selectivity for FM sweeps by calculating the direction (up or down) of sweep that elicits a larger number of spikes in the neuron compared to the other direction(Fuzessery et al., 2006; Gittelman et al., 2009; Kuo and Wu, 2012; Geis and Borst, 2013; Morrison et al., 2018), ignoring key dimensions of the neural response such as spike timing that could encode information about sweep identity. To overcome this problem, we performed in vivo juxtacellular recordings from neurons in the IC of awake, head-fixed mice (Figure 1A–B) and examined direction encoding using both an asymmetry index comparing the number of spikes elicited in each sweep direction and the classification accuracy from a support vector machine (SVM), a supervised machine learning algorithm that was trained on the neuron spike times for each trial. Recordings covered the tonotopic map of the IC with neuron best frequencies (the frequency eliciting the greatest number of spikes at 70 dB SPL) ranging from 4–64 kHz (Figure 1C). To investigate direction selectivity, we played 70 dB SPL logarithmic FM sweeps with speeds of 10 – 200 octaves/second. We observed a diversity of responses, including directionally selective cells (Figure 1D, F), cells that exhibited asymmetries in spike timing between up and down sweeps (Figure 1F), and cells that were inhibited by the sound (Figure 1G). We first evaluated direction selectivity for neurons in our dataset using the direction selectivity index (DSI), which is a normalized comparison of the firing rates of the neuron during the up sweep compared to the down sweep. Neurons with a DSI closer to +1 have higher firing rates in the upward sweep direction, while neurons with a DSI closer to −1 have higher firing rates to the downward direction (Kuo and Wu, 2012). We found that the DSIs of the cells we recorded from formed a continuum (Figure 1I), with most cells being non-selective according to the criteria of Kuo and Wu, 2012 (37/46 cells, defined as DSIs < +0.33 and > −0.33). Of the 9 directionally selective cells, 4 were selective for downward sweeps and 5 were selective for upward sweeps.

However, the DSI measure only considers the total number of spikes occurring during the up or down sweep, disregarding information about spike timing and other mechanisms that could be used by downstream neurons to decode selectivity. To overcome this problem, we trained a support vector machine model (SVM) on binned spike counts from up and down sweep trials to investigate whether the direction of the sweep could be decoded from the spike trains of individual IC neurons (Figure 1G, example shown uses 16 time bins PCA transformed and projected onto the first two components for illustrative purposes.) Using the model, we found that the direction of the FM sweep could be determined significantly above chance in 25/46 neurons (Figure 1H). Interestingly, we found that the group of neurons where direction decoding was significant in the model did not completely overlap with the group of neurons that were deemed directionally selective using the DSI. We found that sweep direction could be decoded from the spiking in 20 cells that had non-selective DSIs, and overall found no significant correlation (r^2^ = 0.01) between the absolute value of neuron DSI and model accuracy (Figure 1I).

We hypothesized that the mismatch between the DSI and model results occurred in part because the timing of spikes, which is assessed by the SVM model but not the DSI metric, is critical for direction decoding. To test this hypothesis, we shuffled the order of the time bins while maintaining class labels and compared the accuracy of this model to the true accuracy (bin order intact) for each neuron. We found a significant decrease in the model accuracy across all the cells when the time bins were shuffled (paired t-test, *t* = 7.0, *p* = 1.0e-8) and there were only 2 cells from which the direction could be still be decoded with shuffled bins compared to 25 when the time bin order was intact, suggesting that the timing of spikes, rather than just the total number of spikes, is important for encoding direction in individual neurons (Figure 1J). In particular, we found that cells that more accurately encoded direction had the largest shifts in accuracy after the time bins were shuffled (Figure 1K,L), suggesting that cells that encode direction well are more likely to use temporal codes. Overall, these results suggest that neurons use a combination of strategies including both the timing of individual spikes and the overall firing rate to encode sweep direction for FM sweeps.

### Direction encoding for four-octave FM sweeps varies across sweep intensities but not speed

**Figure 2.**
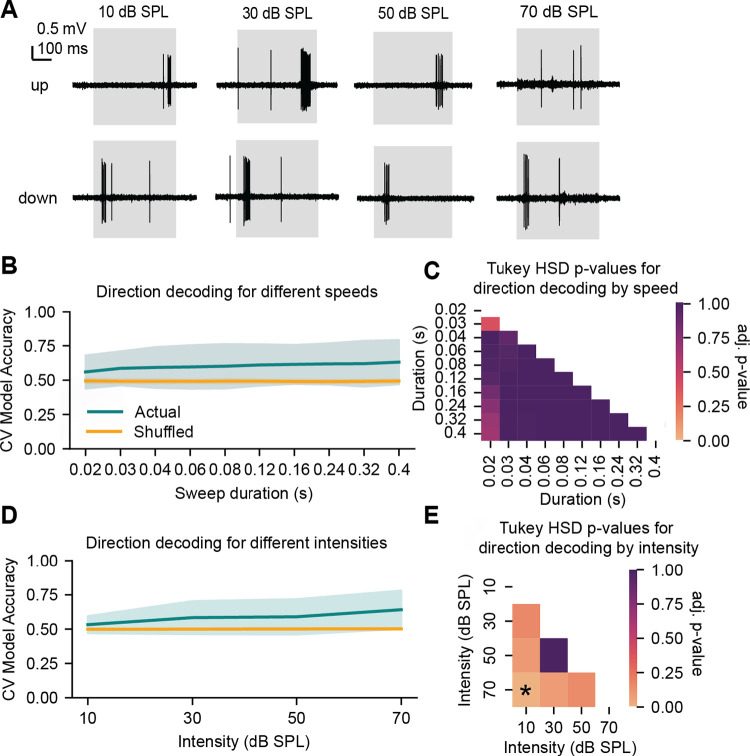
Four-octave FM sweep direction decoding is consistent across varying speeds but not intensities ***A***, Sample traces from a recording showing differing responses to four-octave up and down sweeps (10 octaves/second) at each intensity level. Sound presentation is indicated by the grey box. ***B***, Accuracy of direction decoding across varying sweep speeds. The blue line indicates direction decoding accuracy across speeds for true labels, and the orange line indicates accuracy for shuffled labels (chance). The standard deviation is shown with shading. ***C***, Heatmap showing Tukey HSD p-values for each pairwise comparison of true data accuracy scores for each speed in *B*. Lighter colors indicate more significant results. No comparisons were statistically significant. ***D***, Accuracy of direction decoding across varying sweep intensities. The blue line indicates direction decoding accuracy across intensities for true labels, and the orange line indicates accuracy for shuffled labels (chance). The standard deviation is shown with shading. ***E***, Heatmap showing Tukey’s HSD p-values for each pairwise comparison of true data accuracy scores for each speed in *D*. Lighter colors indicate more significant results. Decoding accuracy at 70 dB SPL was significantly higher than accuracy at 10 dB SPL (Tukey’s HSD, *p* = 0.0001); this is indicated with an asterisk.

We next investigated how neural tuning for sweep direction is affected by changes in sweep speed and sweep intensity. We hypothesized that sweep decoding would be highest at slower sweep speeds, given this would exaggerate the asynchronies in activation of excitatory and inhibitory areas of a neuron’s tonal receptive field that have been suggested to underlie selectivity (Xie et al., 2007; Kuo and Wu, 2012). To examine this, we played upward and downward four-octave FM sweeps with speeds varying from 10 – 200 octaves/second at four different sound intensities (10, 30, 50, and 70 dB SPL, Figure 2A) and used an SVM to decode the sweep direction at each intensity level and each speed. In line with our hypothesis, we found that there was a significant difference in direction decoding across speeds (Figure 2B, repeated-measures ANOVA, *F*(9, 414) = 2.5, *p* = 0.0077). However, post-hoc tests revealed that none of the pairwise accuracies were significantly different from each other (Tukey’s HSD, Figure 2C). In addition, while the number of cells from which direction could be decoded increased as speed decreased, this trend was not significant (Chi-square test for trend, χ^*2*^ = 3,69, *p* = 0.93). These results suggest that IC neurons can robustly encode the direction of four-octave FM sweeps regardless of sweep speed.

Next, we tested whether direction decoding accuracy was also robust across FM sweeps with varying intensities. We hypothesized that decoding accuracy would be highest at the loudest intensity tested (70 dB SPL), as this would again exaggerate spiking differences between inhibitory and excitatory areas on a neuron’s receptive field and lead to greater asymmetries in spike timing between up and down sweeps, which could enhance direction decoding. In addition, IC neuron tonal receptive field shapes form a continuum with many cells exhibiting intensity-dependent changes in frequency tuning (Palmer et al., 2013), suggesting that direction selectivity may also be intensity-dependent. In line with this, we found that direction decoding accuracy in IC neurons differed across varying sweep intensities within individual neurons (Figure 2D, repeated-measures ANOVA, *F*(3, 138) = 12.38, *p* < 0.0001), with decoding accuracy at 70 dB significantly higher than decoding accuracy at 10 dB (Tukey’s HSD, *p* = 0.0001, Figure 2E). In addition, we observed that the number of cells from which direction could be decoded increased as intensity increased (10 dB SPL: 5/46 cells, 30 dB SPL: 14/46 cells, 50 dB SPL: 16/46 cells, 70 dB SPL: 27/46 cells), and this trend was significant (Chi-square test for trend, χ^2^ = 11.97, *p* = 0.007).

Overall, our data show that direction decoding accuracy for four-octave FM sweeps is relatively stable across sweep speed but not sweep intensity, with cells exhibiting higher decoding accuracies at higher intensities.

### Individual IC neurons robustly encode frequency range and speed but not direction of FM sweeps across varying sweep frequency ranges

**Figure 3.**
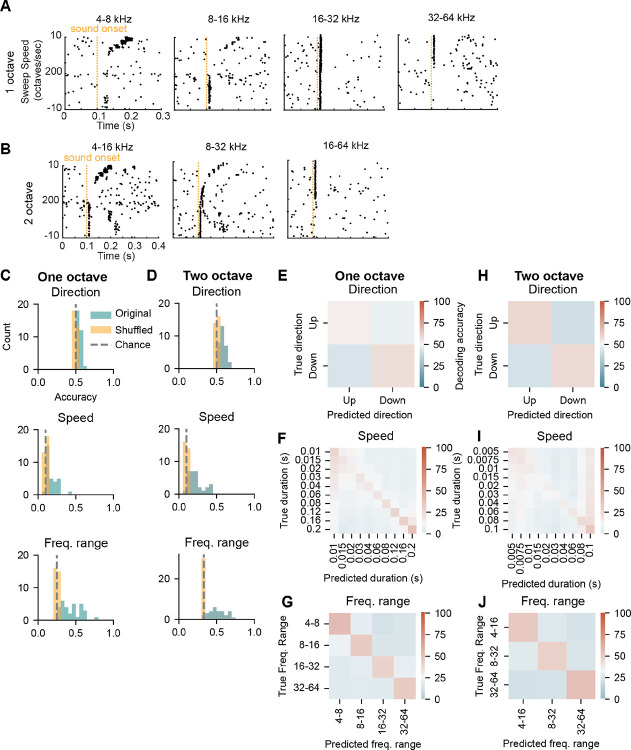
IC neurons encode frequency range, speed of one- and two-octave FM sweeps ***A***, Raster plots with responses to one-octave FM sweeps of varying sweep frequency ranges (listed on top of each plot). Up sweeps occupy the top half of the plot and down sweeps occupy the bottom. Slowest speeds are on the outsides with fastest speeds in the middle. Sound onset is shown by the orange line at 100 ms. ***B***, Raster plots showing responses from the same neuron in *A* to the two-octave FM sweeps, played at the same speeds. ***C***, Direction, speed, and frequency range decoding accuracy for one-octave FM sweeps. True accuracy is shown in blue, accuracy with shuffled bins is in orange. Chance for each plot is shown with a grey dashed line. ***D***, Decoding accuracies for the same sound features shown in *C*, for two-octave FM sweeps. ***E***, Model predictions (x-axis) versus actual (y-axis) categories for decoding of each sound feature. Predictions are aggregated across all neurons in the dataset. Chance performance is white, while above chance is red and below chance is blue. ***F-G***, Same as A, for speed and frequency range decoding for one-octave sweeps. ***H-J***, Same as *E*-*G* for decoding from two-octave sweeps.

Our results support the idea that some IC neurons encode sweep direction for four-octave FM sweeps. However, natural sounds rarely contain FM sweeps with such large frequency changes: many ethologically relevant sounds such as vocalizations have small frequency changes on the order of ½ to 1 octave (Holy and Guo, 2005; Portfors, 2007). Whether the direction of frequency change is encoded in IC neurons for smaller sweeps has been less well studied. To examine whether neurons encode sweep direction across sweeps of varying size, we played both one- and two-octave FM sweeps at speeds of 10 – 200 octaves/second while recording from IC neurons in awake mice. One-octave FM sweeps consisted of the frequency ranges 4–8, 8–16, 16–32, and 32–64 kHz, and two-octave FM sweeps consisted of the frequency ranges 4–16, 8–32, and 16–64 kHz (Figure 3A,B).

To determine whether neurons were directionally selective, we used a support vector machine to decode the direction of the sweep from the neuron spike times. Strikingly, we found that we could only decode direction from 2/31 of the cells in our dataset in the one-octave sweep condition (Figure 3C, top), and in the two-octave condition, direction could only be decoded in 9/30 cells (Figure 3D, top). This finding differed from the four-octave sweep results, where direction was encoded in 25/46 cells, and suggests that selectivity for direction is not a sound feature that is categorically encoded in individual mouse IC neurons for ethologically relevant FM sweep frequency changes.

Instead, we next asked whether other features of the sweep—in particular, the frequencies present in the sweep and the sweep speed—could be decoded from each neuron’s response. In contrast to the direction decoding results, we found that the speed of each sweep could be decoded significantly above chance from most cells in both the one- and two-octave sweep conditions regardless of sweep direction or frequency range (25/31 and 27/30 cells, respectively, Figure 3C,D middle). In addition, we found that the frequency range of the sweep, regardless of sweep direction or speed, could be decoded above chance in most cells in our dataset (31/31 cells for one-octave sweeps and 24/30 cells for two-octave sweeps, Figure 3C,D bottom).

Prediction errors made by the model varied for all three sweep features tested. Decoding accuracy for the direction of one- and two-octave sweeps was near chance performance, with the model predicting the sweep direction incorrectly almost as often as it predicted direction correctly (Figure 3E,H). Interestingly, for the one-octave sweeps the model skewed towards predicting slower sweep speeds, with lower relative accuracy for fast speeds (Figure 3F), and the model tended to predict slower speeds than the actual stimuli for most trials. This contrasted to prediction errors for the two-octave data, where the model had highest accuracy in predicting both the fastest and the slowest sweep speeds, but tended to predict the fastest speed even for trials that were slower (Figure 3J). Sweep frequency range decoding was similarly high across all sweep ranges tested (Figure 3G,J). Our data suggest that individual neurons do not categorically encode the direction of one- and two-octave FM sweeps, but instead provide information about other features of the stimuli, such as the frequency range and speed.

### Individual IC neurons multiplex features of FM sweeps

**Figure 4.**
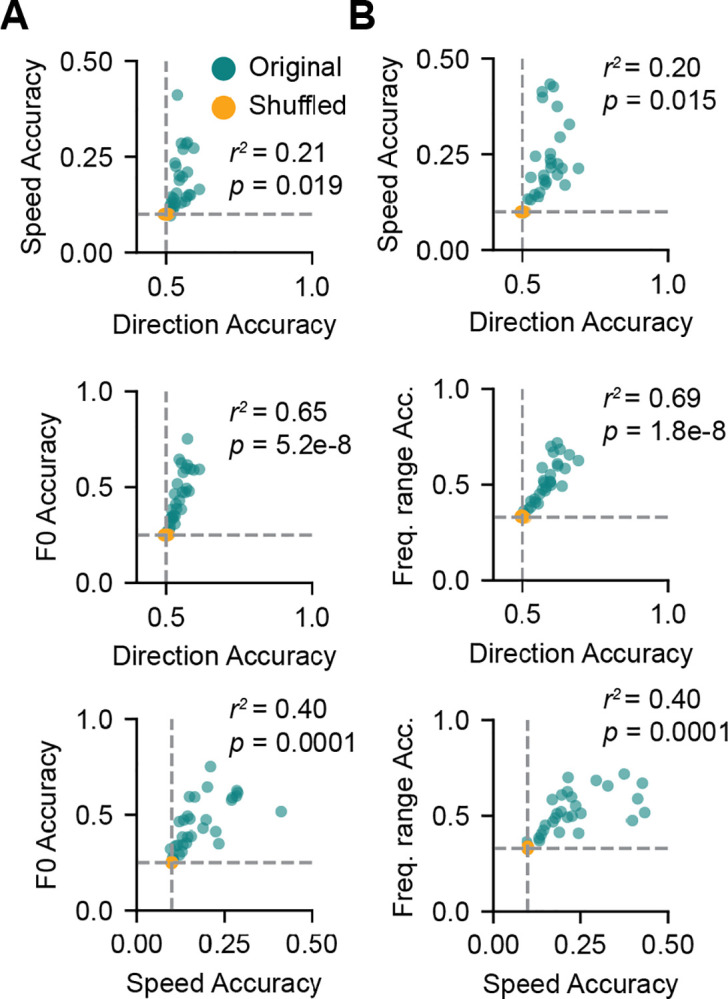
IC neurons multiplex features of one- and two-octave FM sweeps ***A***, Scatterplots showing correlations between feature encoding accuracies in individual cells for one-octave FM sweeps. Models trained on true class labels are shown in blue and models trained on shuffled class labels are shown in orange. R-squared and p-values from linear regressions between true data points for feature pairs are shown on each plot. For six total comparisons, the Bonferroni-corrected significance level (α) = 0.0083. ***B***, Correlations of feature decoding accuracies in individual cells as in *A* for two-octave FM sweeps.

Previous literature suggests that individual IC neurons can jointly encode multiple sound features. For example, IC neurons can multiplex information from multiple sound localization cues with different features of a single spike train (Pena and Konishi, 2002; Chase and Young, 2008). IC neurons can also simultaneously encode visual and auditory information about a sensory stimulus (Schmehl et al., 2025). We asked whether IC neurons that have high encoding accuracy for one sweep feature also have high encoding accuracy for other features of a sweep by correlating the decoding accuracy scores for direction, speed, and frequency range for each cell in our one-octave (Figure 4A) and two-octave (Figure 4B) sweep data sets. We did not find a significant positive correlation between direction and speed accuracies in the one- or two-octave data (α corrected for 6 total comparisons using the Bonferroni correction = 0.0083; linear regression, one octave: *r*^*2*^ = 0.21, *p* = 0.019, two octave: *r*^*2*^ = 0.20, *p* = 0.015). However, direction and frequency range decoding accuracies were strongly correlated in both the one and two octave datasets (one octave: *r*^*2*^ = 0.65, *p* = 5.2e-8; two octave: *r*^*2*^ = 0.69, *p* = 1.8e-8), as were frequency range and speed decoding accuracies (one octave: *r*^*2*^ = 0.40, *p* = 0.0001; two octave: *r*^*2*^ = 0.40, *p* = 0.0001).

These results suggest that many IC neurons can simultaneously encode multiple FM sweep features, such as the frequency range and speed. Multiplexing multiple features of an FM sweep would require a neuron to use different strategies to represent FM sweep features within a single spike train. There is previous evidence for multiplexing in auditory neurons: for example, in cat IC (Chase and Young, 2008) and cat auditory cortex (Furukawa and Middlebrooks, 2002) sound localization cues are encoded using multiple independent spike train features, including first spike latencies and firing rate. In addition, the distribution of inter-spike intervals can also encode information in neurons independent of the sound response PSTH (Bialek et al., 1991; Panzeri et al., 2001; Lundstrom and Fairhall, 2006; Insanally et al., 2019).

**Figure 5.**
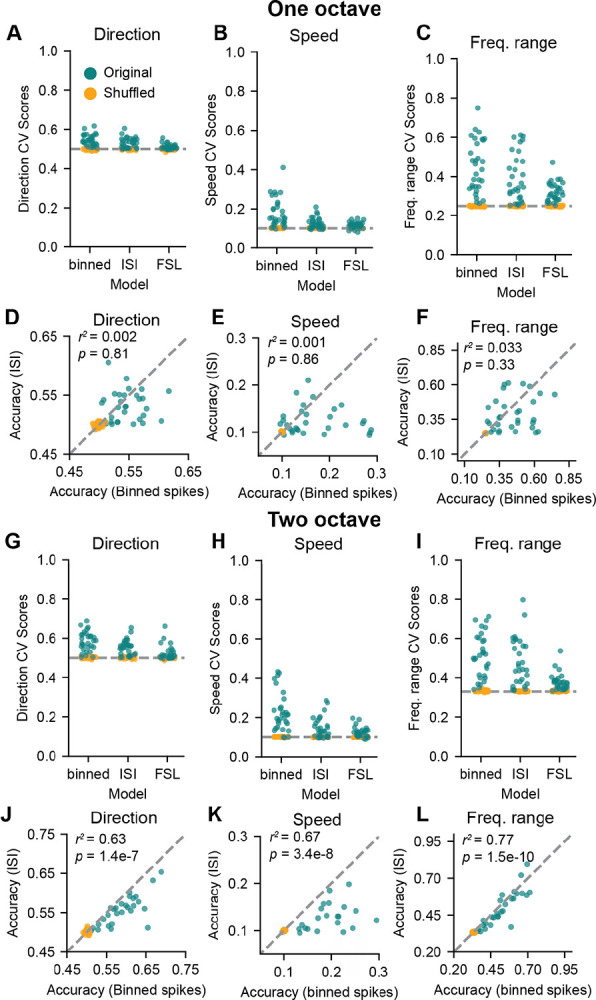
IC neurons use multiple strategies to encode FM sweep features in a single spike train ***A***, Accuracies for decoding direction in one-octave sweeps from models trains on binned spike times, the distribution of inter-spike intervals (ISI), or first spike latency (FSL) in individual cells (blue points) compared to shuffled data (orange points). ***B-C***, Same as *A*, for speed and frequency range. ***D***, Correlation between decoding accuracy in models trained on binned spike times or ISIs for direction in one-octave FM sweeps. ***E-F***, Same as *D*, for speed and frequency range. ***G-L***, Same as *A-F*, for two-octave sweeps.

We investigated whether IC neurons multiplex features of FM sweeps by training the decoder to predict sweep direction, frequency range, and speed from either the binned spike times (as shown previously), distribution of inter-spike intervals, or first spike latency for the trial. For both one and two octave FM sweeps, we found that accuracy scores obtained using the three strategies were not significantly different from each other across the neuron population for decoding direction (Friedman test, one octave: χ^2^(30) = 1.03, *p* = 0.60, Figure 5A, two octave: χ^2^(29) = 0.07, *p* = 0.97, Figure 5G), speed (one octave: χ^2^(30) = 0.06, *p* = 0.96, Figure 5B, two octave: χ^2^(29) = 2.87, *p* = 0.24, Figure 5H) or frequency range (one octave: χ^2^(30) = 0.58, *p* = 0.75, Figure 5C, two octave: χ^2^(29) = 3.20, *p* = 0.20, Figure 5I).

We next investigated whether neurons in our dataset redundantly encode information using both spike timing and inter-spike interval distribution or if some neurons used one strategy over the other. To do this, we correlated decoding accuracies from models trained using each strategy. Interestingly, in the one octave dataset we found that there was no correlation between decoding accuracies for sweep direction (*r*^*2*^ = 0.002, *p* = 0.81, Figure 5D), speed (*r*^*2*^ = 0.001, *p* = 0.86, Figure 5E), or frequency range (*r*^*2*^ = 0.033, *p* = 0.33, Figure 5F), or speed This suggests that neurons tend to use one strategy or the other to encode these features rather than both, and we found neurons that were strong encoders in our dataset for both codes relying on spike timing and those relying on inter-spike interval.

In contrast to results from the one-octave dataset, decoding accuracies from models trained on binned spike times and inter-spike intervals were highly correlated in the two-octave dataset for direction (*r*^*2*^ = 0.63, *p* = 1.4e-7, Figure 5J), speed (*r*^*2*^ = 0.67, *p* = 3.4e-8, Figure 5K), or frequency range (*r*^*2*^ = 0.77, *p* = 1.5e-10, Figure 5L). This difference in decoding strategies for one- and two-octave data may be suggestive of differences in how neurons encode FM sweep information over time: given the shorter one-octave stimulus, information about sweep features may be present in only one spike train metric, whereas the longer two-octave stimulus may yield redundancy in coding strategies over the time course of the sound. In addition, the mean decoding accuracy was higher for all sweep features in the two-octave dataset. The increased correlation in decoding accuracies for two-octave sweeps may emerge from the greater time window over which information can be encoded by each neuron. Overall, these data suggest that IC neurons multiplex information about FM sweeps using different strategies that include the spike timing, distribution of inter-spike intervals, and first spike latency.

### Temporal cues are critical for encoding sweep speed but not sweep frequency for one- and two-octave FM sweeps

We previously showed that IC neurons encode sweep direction for four-octave sweeps in a timing-dependent manner, such that disrupting the temporal information contained in the spiking pattern while leaving the overall number of spikes intact significantly decreased the direction decoding accuracy (Figure 1K,L). This suggests that temporal cues are critical for how IC neurons convey stimulus-specific information for FM sweeps. We next asked whether temporal cues are also important for how IC neurons encode frequency range and speed for one- and two-octave sweeps, since these can be decoded above chance in many neurons (Figure 3C,D). To examine this, we trained an SVM on the spike time data for one- and two-octave FM sweeps. We then compared performance of the decoder with the time bins intact vs. with the time bins for each trial in a shuffled order, which disrupted the temporal information in each trial while preserving the total number of spikes.

**Figure 6.**
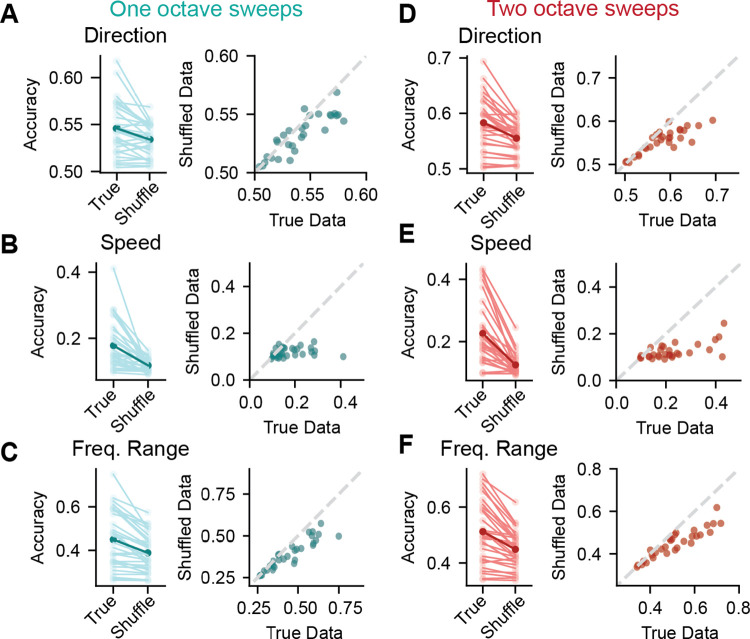
Spike timing is a critical component of feature encoding for one- and two-octave FM sweeps ***A***, Left panel: comparison of accuracy scores for decoding sweep direction for one-octave FM sweeps across each neuron where the bin order is intact (True) and shuffled (Shuffle). Class labels are maintained by trial in both cases. The mean is shown in dark blue, and light blue lines represent individual neurons. Right panel: scatterplot comparing accuracy with true bin order and shuffled bin order in individual neurons. The line of identity is shown in dashed grey (x = y). ***B-C***, same as *A* for speed and frequency range encoding across true and shuffled bin data for one-octave FM sweeps. ***D-F***, same as *A-C* for two-octave FM sweeps.

We found that shuffling the order of the time bins in the one-octave condition significantly decreased the accuracy of the model for sweep direction (Figure 6A, paired t-test, *t* = 3.93, *p* = 0.0005), speed (Figure 6B, *t* = 4.61, *p* = 7.0e-5), and frequency range (Figure 6C, *t* = 5.62, *p* = 4.04e-6). In line with this, there were also fewer cells from which direction and speed could be significantly decoded when the bins were shuffled (direction: 0 shuffled vs. 2 unshuffled, speed: 13 shuffled vs. 25 unshuffled), but the number of cells that encoded frequency range significantly above chance changed only minimally when the bins were shuffled (29 shuffled vs. 31 unshuffled), even though mean decoding accuracy decreased. A similar result was found in the two-octave condition, where shuffling the order of the time bins also significantly decreased the accuracy of the model for direction (Figure 6D, *t* = 5.19, *p* = 1.51e-5), speed (Figure 6E, *t* = 6.59, *p* = 3.22e-7), and frequency range (Figure 6F, *t* = 5.46, *p* = 7.08e-6). Again, we found that the number of cells that encoded speed and direction above chance decreased in the shuffled condition (direction: 1 shuffled vs. 9 unshuffled, speed: 11 shuffled vs. 27 unshuffled). However, despite a decrease in accuracy in the shuffled condition, the number of cells that encoded frequency range above chance remained the same in both conditions (24 shuffled vs. 24 unshuffled).

These results suggest that spike timing may be more important for conveying sweep speed compared to sweep frequency range, as frequency range decoding accuracy was less affected by timing disruption. These findings further support the idea that IC neurons can multiplex information by using different coding strategies to represent different sound features.

### IC neurons encode sound features across multiple time scales

Previously, decoding algorithms were trained on all the spiking information from a neuron across an individual trial, such as the binned spike times or distribution of inter-spike intervals across hundreds of milliseconds. However, postsynaptic targets are constrained in their ability to integrate inputs across time by the passive and active membrane properties of the target neurons. While this could be seen a coding limitation, this constraint would allow presynaptic neurons to encode different sound features at different times during the response, which could then be read out by one or multiple postsynaptic neurons with varying temporal integration windows.

**Figure 7.**
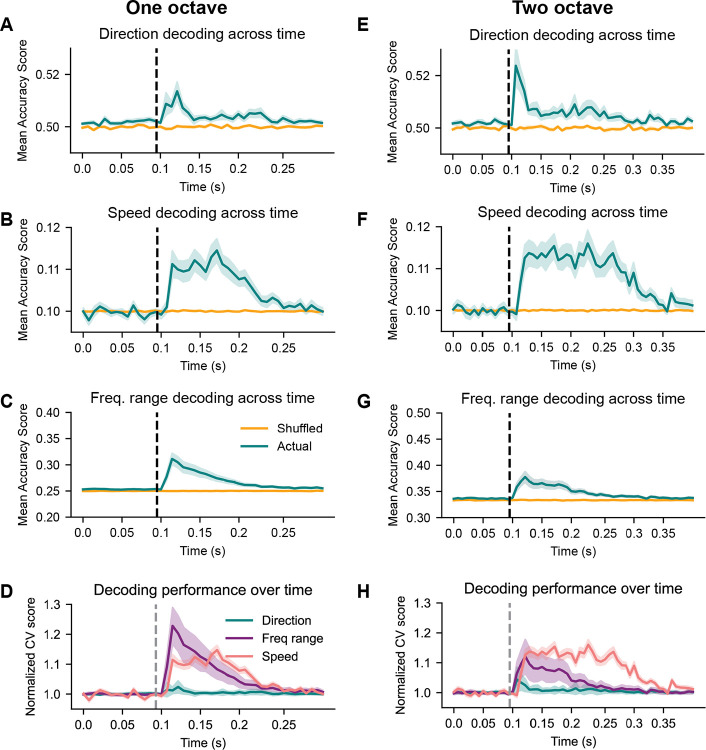
IC neurons encode FM sweep features over different time scales relative to stimulus onset ***A***, Direction decoding accuracy across time (6.9 ms time bins) for one-octave FM sweeps. The blue line indicates decoding accuracy for data with true labels, and the orange line indicates decoding accuracy for data with shuffled labels. Shading around each line represents the standard error of the mean. The dashed black line marks stimulus onset (100 ms). ***B-C***, Same as *A* with speed and frequency range decoding across time for one-octave FM sweeps. ***D***, Overlay of accuracy scores from *A-C* normalized to their relative pre-stimulus values. ***E-G***, Same as *A-C* for two-octave stimuli. ***H***, Same as *D* for two-octave stimuli.

To investigate this question, we trained an SVM to decode the direction, speed, and frequency range of an FM sweep using consecutive 6.9 ms windows (3 combined time bins) of each neuron’s response to one-octave FM sweeps of varying directions, speeds, and frequency ranges. We found that direction decoding was relatively poor throughout the duration of the sound but peaked at the beginning of the stimulus (Figure 7A), an effect likely due to strong onset or offset spiking for one sweep direction in some cells. Decoding accuracy for sweep speed occurred later compared to stimulus onset but remained high throughout the duration of the sound (Figure 7B), whereas frequency range decoding increased rapidly after the onset of sound and then decayed (Figure 7C, compared in Figure 7D). Direction decoding was better for the two-octave sweeps compared to the one-octave sweeps and peaked at the stimulus onset (Figure 7E). Decoding across time was similar for the other features of the two-octave FM sweeps, with consistent decoding of speed throughout the duration of the stimulus (Figure 7F) and a fast increase in decoding accuracy for frequency range (Figure 7G) at the beginning of the stimulus.

We next asked how the accuracy for encoding each feature differed in time compared to chance level. To do this, we normalized each accuracy score to the baseline pre-stimulus accuracy (at chance level) and compared decoding accuracy over time for each feature. For both one- and two-octave sweeps, we found that accuracy for frequency range increased first and then declined, while speed decoding increased later than frequency range but was consistent throughout the duration of the stimulus (Figure 7D,H, pink and purple lines). Relative to chance, decoding accuracy for direction was poor across all time bins compared to the accuracy values achieved for frequency range and speed (Figure 7D,H blue lines).

These results suggest that individual IC neurons can represent sound features across varying time scales, which could provide another mechanism for downstream decoders to receive information about complex stimuli.

### Populations of IC neurons provide robust representations of FM sweep features

While we found that decoding sweep frequency range and speed from individual neurons was above chance in many cells, accuracy values were rarely high enough to provide high-confidence information about the stimulus (Figure 3). However, the response heterogeneity and multiple coding strategies used by individual IC neurons could comprise a population code for various sound features. Population representations of other sound features, including sound location and amplitude-modulated stimuli, have been previously shown in IC neurons (Zohar et al., 2013; Day and Delgutte, 2016; Rogalla et al., 2024; Shi et al., 2024), but population codes for FM sweep features have not been investigated. We examined the population benefit for encoding the speed, frequency range, and direction of one- and two-octave FM sweeps by grouping cells into pseudo-populations that consisted of 2–21 neurons and compared the decoding accuracy for these groups to the accuracy obtained from single neurons.

**Figure 8.**
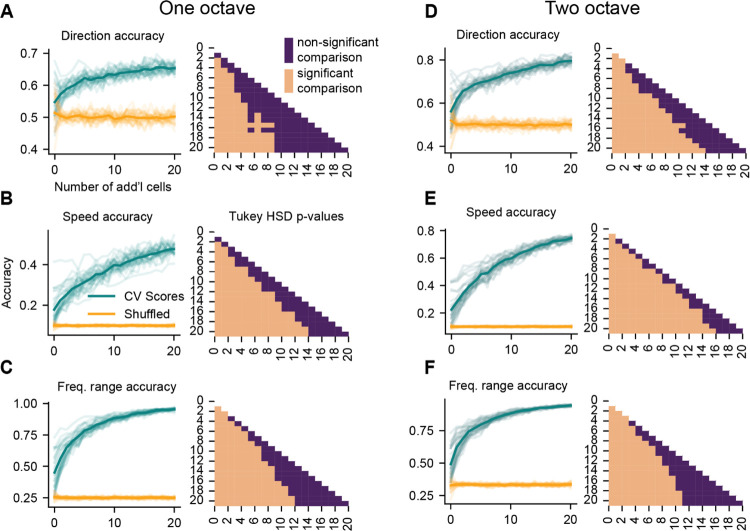
Pseudo-populations of IC neurons provide higher decoding accuracy compared to individual neurons ***A***, Left: Direction decoding accuracy of each neuron in the one-octave dataset paired with 1–20 additional neurons. Each light blue line represents decoding from one seed neuron, and the dark blue line represents the mean at each pseudo-population size. Orange lines show accuracy scores from decoding with class labels shuffled. Right: Tukey’s HSD post-hoc test corrected p-values from pairwise comparisons between the true accuracy of each pseudo-population size. Light squares represent significant comparisons while dark squares indicate comparisons that were not significantly different from each other. ***B-C***, Same as *A* for speed and frequency range decoding. ***D-F***, Same as *A-C* for two-octave FM sweeps.

In the one-octave dataset, we found that increasing the number of neurons in the pseudo-population (starting with decoding from individual cells) significantly increased the decoding accuracy for direction (Figure 8A*, F*(20,630) = 58.85, *p* < 0.0001), speed (Figure 8B repeated measures ANOVA, *F*(20,630) = 141.8, *p* < 0.0001), and frequency range (Figure 8C, *F*(20,630) = 263.64, *p* < 0.0001). As cells were added to the population, direction decoding accuracy reached an asymptote that approached 65% accuracy when the population contained approximately 15 cells (Figure 8A). Speed decoding accuracy increased nearly linearly with each cell added to the population, and decoding accuracy was limited by the number of neurons in our dataset and thus the number of cells that could be added to the population (Figure 8B). Strikingly, frequency range decoding approached 100% accuracy and did not significantly increase after the pseudo-population contained 16 cells, suggesting this population size could reliably convey frequency range information for one-octave FM sweeps (Figure 8C).

Population decoding accuracies from the two-octave datasets largely mirrored those from the one-octave results. Increasing the number of cells in the pseudo-population significantly increased the decoding accuracy for direction (Figure 8D, *F*(20, 609) = 137.74, *p* < 0.0001), speed (Figure 8E *F*(20, 609) = 452.70, *p* < 0.0001), and frequency range (Figure 8F, *F*(20, 609) = 290.97, *p* < 0.0001). Again, direction decoding accuracy was better in the two-octave dataset compared to the one-octave dataset, and decoding accuracy began to plateau around 15 cells at a decoding accuracy of 80% (Figure 8D). Speed decoding accuracy increased with the number of neurons in our dataset, and the trend in the data suggests that increasing the pseudo-population size would have continued to increase decoding accuracy (Figure 8E). In addition, frequency range decoding again approached 100% at a pseudo-population size of approximately 16 neurons (Figure 8F).

Overall, these results show that population codes convey more accurate information about the features of FM sweeps than individual IC neurons.

### Frequency change direction for vocalizations is not encoded in individual IC neurons

**Figure 9.**
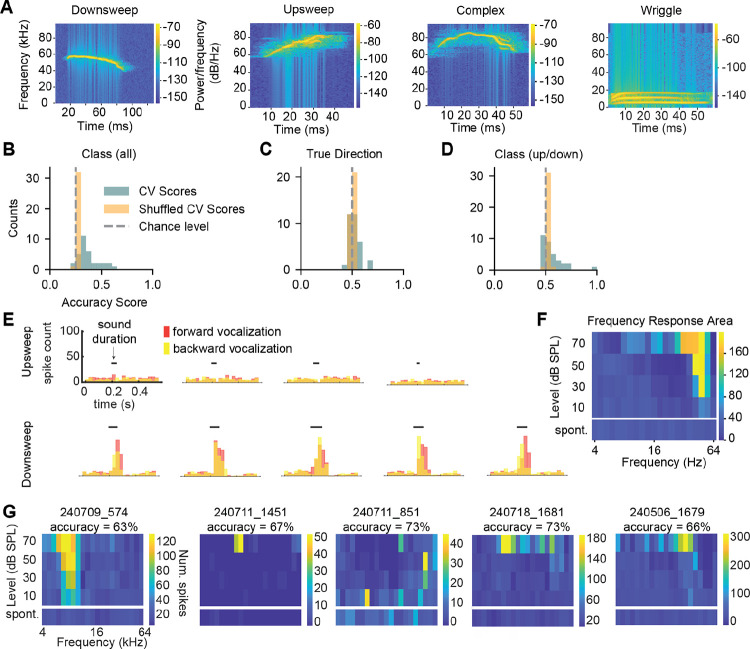
IC neuron responses to vocalizations do not depend on frequency sweep direction ***A***, Spectrograms of four example vocalizations (one from each class) used as stimuli. ***B***, Decoding accuracy for vocalization class in individual IC neurons (blue) compared to decoding from shuffled classes (orange). Chance is labeled with a grey dashed line at 25%. ***C***, Decoding accuracy for the true direction of the frequency sweep contained in the vocalization. ***D***, Decoding accuracy for the vocalization class regardless of whether the stimulus was played forward or backward. ***E***, Example peristimulus time histograms for the response from one neuron to each of the example upsweep vocalizations and downsweep vocalizations. Response to the forward vocalization is in pink and response to the backward vocalization is in yellow. The black bars indicate the sound presentation window. ***F***, The frequency response area for the neuron in *E*, constructed from responses to 5 presentations each of 200 ms pure tones at 4 intensities and over 4 octaves with 5 frequency steps/octave. Spontaneous firing is shown in the bottom row. ***G***, Frequency response areas from neurons where vocalization type (up/down) could be decoded significantly above chance.

Both human speech and mouse vocalizations contain rapid frequency sweeps (Portfors, 2007), and previous studies have shown that some IC neurons fire selectively for specific vocalizations (Portfors et al., 2009; Mayko et al., 2012; Lawlor et al., 2023). However, whether the frequency sweeps present in conspecific vocalizations shape individual neuron selectivity is not well understood. To examine this, we recorded from IC neurons while playing four types of calls (calls from Schiavo et al., 2020; Valtcheva et al., 2023): an ultrasonic upsweep, an ultrasonic downsweep, an ultrasonic vocalization with complex (up and down) frequency changes, and a call with multiple low-frequency bands (in order, we refer to these as upsweep, downsweep, complex, and wriggle, Figure 9A).

Consistent with previous studies, we found that some neurons exhibited selective responses for specific vocalization classes. To quantify how well individual neurons encoded vocalization class, we trained an SVM on the spike trains of individual neurons. The SVM predicted the vocalization class above chance (25%) in 17/33 cells (Figure 9B), with an average accuracy of 44% in cells with significant encoding. To investigate whether the direction of the FM sweep within the vocalization was important for vocalization discriminability, we next performed decoding on two sets of responses. For the first model, we grouped responses for vocalizations where there was a true upsweep (upsweeps, and downsweeps played backwards) and trained the decoder to discriminate these from responses where there was a true downsweep (downsweeps, and upsweeps played backwards). Interestingly, we found that in only 3/33 cells in our dataset could the direction of the vocalization be decoded significantly above chance (Figure 9C), and average accuracy for cells with significant decoding was 62%, suggesting that most IC neurons do not specifically encode the direction of frequency sweeps contained within vocalizations.

We next asked whether the upsweep or downsweep vocalization class could be decoded using the neuron spike times regardless of whether the vocalization was played forwards or backwards. Here, we found that 7/33 neurons could discriminate the class of the sweep, and these neurons had higher accuracy (72%) compared to the decoding for the true direction (Figure 9D). We examined the peri-stimulus time histograms (PSTHs) for the upsweep and downsweep vocalizations in the neuron with the strongest decoding accuracy and found exclusive responses to the downsweep vocalization regardless of what direction it was played (Figure 9E), indicating that information other than the direction of the frequency change is important for vocalization class selectivity in this neuron. In addition, we found that in this highly selective neuron, the tonal receptive field overlapped with the frequency content of the preferred vocalization (Figure 9F). However, this was not always the case: we obtained frequency response areas (FRAs) from 6/7 neurons where vocalization class (up/down) could be decoded significantly above chance and found that only two of them exhibited clear V-shaped tuning. In one of these cells (240709_574), the preferred frequency was around 10 kHz, which was much lower than the frequencies present in either vocalization (Figure 9G). The FRAs from the remaining four neurons exhibited some tuning at higher intensities, but again tuning did not overlap with the vocalization spectral content. These results indicate that vocalization responses in the mouse IC are likely generated through neurons with mixed selectivity for multiple features of the sweep as compared to simple tonal or directional receptive fields.

## Discussion

Here, we used machine learning models to examine how multiple sound features of an FM sweep, including the frequency range, speed, and direction, are encoded in the spike trains of individual IC neurons. We found that single IC neurons can multiplex FM sweep features, and neurons often use multiple coding strategies simultaneously, including temporal patterning of spikes, firing rate, inter-spike interval, and first spike latency. In addition, we showed that IC neurons can encode sound features at different time points during a stimulus, providing an additional mechanism for multiplexing. We also examined decoding in pseudo-populations of IC neurons and found that, when combined, the heterogeneous responses of single neurons can yield robust population encoding of FM sweep sound features. Lastly, we showed that the directionality of frequency sweeps has little influence on vocalization selectivity in single IC neurons, indicating that vocalization selectivity may be better predicted by the receptive fields generated in individual neurons for other sound features.

### Machine learning methods capture alternative coding strategies used by IC neurons

Many previous studies in the IC have investigated neuron selectivity for FM sweep features using selectivity indices such as the DSI, a normalized measure that compares the total number of spikes elicited by an up sweep to those elicited by a down sweep (e.g., Andoni et al., 2007; Williams and Fuzessery, 2010; Kuo and Wu, 2012; Geis and Borst, 2013). Tonal receptive fields that describe frequency selectivity in individual IC neurons are also typically constructed by comparing the total number of spikes elicited by different sound frequencies. However, total spike counts are only one way that neurons may encode sound features, and as sounds often have rich temporal structures, evaluating selectivity using only summed spike counts may omit temporal and other cues that could contain stimulus-encoding information. Evaluating selectivity in this way also assumes that downstream neurons are decoding information by acting as simple linear summators, but a plethora of electrophysiological evidence and computational modeling suggests that this is not the case. For example, dendrites alone have numerous mechanisms that support non-linear integration (for example, Tamás et al., 2002; Gómez González et al., 2011; see Palmer, 2014 for a review).

Here, we instead used a support vector machine to predict sound features from spike times, a method which utilizes temporal information with high resolution (here, 2.3 ms). We found that the direction of four-octave FM sweeps could be decoded from the spiking of many more cells than would be predicted from the DSI. In addition, we showed that disrupting spike timing in the model while maintaining the same number of spikes for each trial significantly reduced the decoding accuracy for direction in four-octave sweeps. It also significantly reduced the decoding accuracy for direction, sweep speed, and frequency range in one- and two-octave sweeps. These results indicate that there is information conveyed in spike timing about not only the direction of FM sweeps but also other sweep features, and this information is lost using methods that disregard when spikes occur in time.

### IC neurons multiplex sound features using several strategies

We highlight the temporal patterning of spikes as one way that neurons can encode the direction of FM sweeps, but we also found that IC neurons use several other strategies to encode stimulus-specific information. In particular, we found that the distribution of inter-spike intervals and first-spike latency contain stimulus-specific information for one- and two-octave sweeps (Figure 5). Previous studies in auditory cortex suggest that individual neurons can use many of these strategies independently of each other (Insanally et al., 2019), providing a mechanism by which neurons can multiplex features of sounds. Here, we found that IC neurons multiplex features of FM sweeps, including the frequency range and speed Figure 4). These data are supported by other work demonstrating joint encoding in IC neurons, for example, with encoding of sound localization cues (Chase and Young, 2008), mixed representations of sound and task variables during a behavioral task (Quass et al., 2024), and dual encoding of auditory and visual stimuli (Schmehl et al., 2025). Ultimately, a critical step toward understanding how IC neurons encode information will be to compare the encoding strategies used in the IC and the decoding strategies used by postsynaptic targets, especially in the medial geniculate body (MGB), the primary ascending target of the IC. To date, there have been few studies investigating how MGB neurons decode information from the IC, and this is an area of future research that is critical for understanding how sound information is represented along the ascending auditory pathway.

### IC neurons exhibit mixed selectivity in response to complex sound stimuli

A common approach in past studies of the IC has been to categorize IC neuron responses by their selectivity for (or tuning to) specific sound features. However, emerging evidence from analyses of large neural datasets suggests that neuron responses are rarely truly categorical (Raposo et al., 2014; Posani et al., 2024). Here, we found that individual IC neurons exhibited mixed selectivity with respect to sweep direction, with direction selectivity co-varying with sweep speed and intensity (Figure 2). Mixed selectivity has also been previously shown for sound and task-dependent variables in some IC neurons (Quass et al., 2024) and for features of FM sweeps (Andoni et al., 2007; Andoni and Pollak, 2011).

Here, we also show that even in neurons that encoded FM sweep direction, this alone did not inform how those neurons responded to a vocalization with similar directional changes (Figure 9). In addition, categorical neuron responses may not be necessary for robust population codes to exist within the IC. In neocortex, a recent study suggests that categorical responses typically underlie low-dimensional rather than high-dimensional population responses (Posani et al., 2024). Similarly, studies in the IC suggest that neuron population codes for amplitude-modulated stimuli do not depend on neurons with high selectivity (Shi et al., 2024), and correlations generated between categorical neurons constrain rather than enhance the amount of information that can be contained in IC neuron populations (Zohar et al., 2013). In line with this, we found that decoding accuracy from single neurons for sound features was on average quite low (Figure 3), and we did not find evidence for cells that encoded direction robustly across changes in differing frequency ranges and speeds. However, pseudo-population decoding accuracy was high despite this (Figure 8) and increased with increasing neurons in the pseudo-population, pointing to robust population coding for FM sweep features in the IC.

Overall, our data suggest that mixed selectivity and multiplexing in individual IC neurons provide a powerful framework for understanding neural coding in the IC. An important next step for future studies is to determine how the MGB and other brain regions that receive input from the IC use the representation of complex sound features that arises from multiplexing in IC neurons.

## Figures and Tables

**Table 1.** Stimuli used for in vivo electrophysiological recordings

Stimuli	Frequency range (kHz)	Intensity (dB SPL)	Speeds (octaves/sec)	Repeats
1 octave FM sweeps	4–8 8–16 16–32 32–64	70	10–200	10
2 octave FM sweeps	4–16 8–32 16–64	70	10–200	10
4 octave FM sweeps	4–64 at 5 steps/octave	10, 30, 50, 70	10–200	5
Pure tones (200 ms)	4–64 at 5 steps/octave	−100, 10, 30, 50, 70	N/A	5
Vocalizations	N/A	70 dB	N/A	10

Table 1. Stimuli used for in vivo electrophysiological experiments.

**Table 2.** Idealized bin sizes and number of bins used for analysis.

Stimuli	Stimulus duration (ms)	Binned duration (stimulus + 20%)	Num. bins that resulted in highest decoding accuracy	Bin size (ms) that resulted in highest decoding accuracy	Num. bins used in analysis	Bin size (ms) used in analysis
4 octave FM sweeps	400	480	55	8.7	209	2.3
2 octave FM sweeps	200	240	75	3.2	104	2.3
1 octave FM sweeps	100	120	51	2.3	51	2.3

Table 2. Bin sizes and number of bins used for analysis. We first calculated the idealized bin sizes for machine learning model. To determine the best bin size to use for the decoding model, we trained the SVM to decode direction in 1, 2, and 4 octave sweeps from spike time data binned using 1–501 bins in steps of 2 bins. The listed values are the bin sizes that yielded the highest direction decoding accuracy for each stimulus. Next, we calculated the number of bins needed for each stimulus to maintain a consistent time window (2.3 ms) over which each of the trials were binned. Because the 1, 2, and 4 octave stimuli were different lengths (sweep speeds were conserved across stimuli), this resulted in a different number of bins for the 4, 2, and 1 octave FM sweep stimuli.

## Data Availability

All data will be made available upon reasonable request to the corresponding author.
